# Biofertilizer and NPSB fertilizer application effects on nodulation and productivity of common bean (*Phaseolus vulgaris* L.) at Sodo Zuria, Southern Ethiopia

**DOI:** 10.1515/biol-2022-0537

**Published:** 2023-01-24

**Authors:** Melkamu Dela, Dereje Shanka, Dawit Dalga

**Affiliations:** Rural Community Based Development Initiative Association, Wolaita Sodo, Ethiopia; College of Agriculture, Wolaita Sodo University, P.O. Box 138, Wolaita Sodo, Ethiopia

**Keywords:** blended fertilizer, inoculation, *Rhizobia etli*

## Abstract

Common bean is among the vital legumes cultivated for nutrition, foreign currency earnings, and income generation. Common bean production is constrained mainly by nutrient deficiencies. A field experiment was conducted at Sodo Zuria Woreda to assess the effects of blended N–P–S–B fertilizer rates and Rhizobium strain on yield and yield traits of common bean and income of farmers. Treatments were five different rates of blended NPSB fertilizer (0, 50, 100, 150, and 200 kg ha^−1^) and two types of Rhizobium strains (HB-A15 and HB-429 (*Rhizobia etli*)) and control (without inoculation). Randomized complete block design in factorial arrangement with three replications were used. Results showed that interaction of blended NPSB rate and Rhizobium strain type had significant effect on the number of effective nodule, grain yield, and above ground dry biomass. Application of 150 kg NPSB ha^−1^ along with inoculation of *Rhizobium etli* resulted in the highest grain yield (3017.7 tons ha^−1^). Similarly, economic analysis indicated that the highest net return of 38298.545 ETB ha^−1^ was obtained from combined application of 150 kg NPSB ha^−1^ along with *Rhizobium etli*. Thus, it could be concluded that using *Rhizobium etli* strain with 150 kg NPSB ha^−1^ was found to be appropriate for common bean production in the area.

## 1 Introduction

Common bean (*Phaseolus vulgaris* L.) is the most important calorie and protein source for humans [1]. In 2012, 23.9 million tons of dry bean, 20.7 million tons of green bean, and 1.9 million tons of string or common bean were projected to have been produced globally [2]. Common bean is one of Ethiopia’s most important grain legumes, with production centered on small-holder farms where nitrogen (N) fertilizer use is limited and average yields are low, typically less than 1 ton ha^−1^ [3]. It is one of the fastest-growing legume crops in Ethiopia, providing an important portion of the daily nutrition as well as foreign earnings for most Ethiopians [4]. It is one of the fast expanding legume crops that provide an essential part of the daily diet and foreign exchange earnings for most Ethiopians [4].

However, the present national average yield of common bean (1.48 tons) is significantly less than the yield that most improved varieties can achieve under good management conditions (2.5–3.0 tons ha^−1^). Poor agronomic techniques, such as insufficient soil fertility management, lack of superior varieties for the different agro-ecological zones, and untimely and inappropriate field operations, could be the major contributing factors for low yield of the crop in the country [5]. In addition to N and P deficiency, a soil fertility mapping effort in Ethiopia has identified K, S, B, and Cu deficiency in key Ethiopian soils [6]. Furthermore, critical soil nutrients such as N, P, K, S, B, and Cu were found to be inadequate in the research areas’ soils. Some of the nutritional deficits were site specific [7]. As a result, the use of a tailored and balanced fertilizer application can help to address the problem.

Furthermore, leguminous crops, such as common bean, require certain Rhizobium strains for optimal nodule development and nitrogen fixation [8]. Nutritional imbalance and poor nodulation are two of the most significant issues in the production of mung bean [9]. As a result, legume crops should be infected with appropriate bacteria to maximize output and enhance soil quality [10].

There have also been notable research findings in Ethiopia and elsewhere in the world that indicate a significant positive effect of single application of blended fertilizers or application along with inoculation with appropriate Rhizobium species on pulse crop growth, nodulation, yield, and yield attributing traits, including common bean [9,11,12]. For instance, Merkebu [9] found a significant (*P* < 0.05) interaction effect of blended NPSZn fertilizer types with Rhizobium strains on mung bean specific leaf area, leaf area ratio, relative growth rate, above ground biomass, root dry weight, plant height, branch number, tap root length, nodule number, nodule diameter, pod number per plant, and grain yield. Lake and Jemaludin [13] also reported that nodule number and nodule dry mass of common bean were significantly affected by various levels of NPSZnB applications. However, there is hardly any evidence in literature on the effect of blended NPSB and Rhizobia strains on common bean growth and production in Sodo Zuria Woreda. Hence, this study was initiated with the following specific objectives:

- To evaluate the effect of Rhizobia inoculation and blended NPSB on yield and yield components of common bean.
- To assess economically appropriate rates of blended NPSB fertilizer and Rhizobium type for production of common bean in Sodo Zuria Woreda

## 2 Materials and methods

The experiment took place in Sodo Zuria Woreda Offa-sere kebele, Wolaita Zone, Southern Ethiopia, during the 2019 rainy season. The experimental site is 4 km south of Sodo town and at an elevation of 1,812 m above sea level, at 6° 47′ 31.55″ N latitude and 37° 45′ 08.76″ E longitude. The site receives 1,484 mm of yearly rainfall and average annual minimum and maximum temperatures of 16.8 and 21.2°C, respectively. Rainfall for the main growing season begins after June and ends in the last week of September at the experimental location. The experimental field had previously been planted with taro and had never received any fertilizer.

### 2.1 Soil sampling and analysis

Before planting, composite (0–30 cm) soil depth samples were taken in a zig-zag fashion from several locations within the experimental field. Soil pH was measured potentiometrically in 1:2:5 soil–water suspensions with standard glass electrode pH meter [14]. The Walkley and Black [15] method was used to determine organic matter content of the soil. Total N in the soil was determined by the Kjeldahl method [16]. Available soil Phosphorus was determined using the Olsen extraction method as described by Olsen et al. [17]. Available sulfur was analyzed by turbidimetric method [18]. The Bouyoucos hydrometer method [19] was used to determine the soil texture. Electro-conductivity (EC) was determined by standard glass electrode using EC meter. Soil cation exchange capacity (CEC) was determined by ammonium acetate method [20].

### 2.2 Description of experimental materials

Nasir, a popular common bean variety released in 2003, was used in this study. Nasir is an indeterminate semi-climbing plant that takes 95–110 days to reach physiological maturity and has dark-red seed coat pigmentation. It is generally utilized for domestic consumption and has a seeding rate of 90–100 kg ha^−1^ [21]. As a source of N, P, S, and B nutrients, a blended NPSB fertilizer containing 18.7, 37.4, 6.9, and 0.25% was received from the Sodo Zuria Agricultural Office. Rhizobia strains HB-A15 and HB-429 were utilized for this research. HB-429 was first isolated by Hawassa University from common bean root nodule around Adilo in Hayia Zone, which is located in Southern Ethiopia, while HB-A15 was isolated by Holeta Agricultural Center. Sequencing was done for HB-429 and it is found to be *Rhizobium* e*tli*, while HB-A15 was not sequenced. Both species tested and proved to fix atmospheric N by N_2_ Africa in collaboration with Hawassa University. The Menagesha biotech industry, which is located in Addis Ababa, is commercializing the strains.

### 2.3 Treatments and experimental design

The experiment included five levels of blended NPSB fertilizer rates (0, 50, 100, 150, and 200 kg NPSB ha^−1^), two Rhizobia strains (HB-A15 and HB-429 (*Rhizobium etli*)), and no inoculation. There were a total of 15 treatments replicated 3 times and the total treatment will be (5 × 3 × 3 = 45). The experiment was set up in a factorial layout with a randomized complete block design and was replicated three times. Each plot was 2.40 m × 2.30 m (5.52 m^2^) and was divided into six rows of three meters each, with the center four rows serving as a net plot. Plots and adjacent replications were separated by 0.5 and 1 m, respectively. The spacing between plants was 10 cm and spacing between rows was 40 cm. The inoculation of Rhizobia strains was done before sowing and blended NPSB fertilizer was applied according to the treatments at the time of sowing.

### 2.4 Agronomic practices

The experimental field was cleansed of undesired items and plant remnants, and was leveled. The experimental field was then plowed three times by oxen before being planted in early May. A sachet of bio-fertilizer containing 125 g was administered according to the advice, which was 10 g kg^−1^ seeds for inoculation. Before inoculation, seeds were soaked in water for 30 min. Then, excess water was removed from the seeds by placing in a sack. There after sugar was applied as adhesive material to stick the inoculums in to the seeds. Finally, the inoculums were applied to the seeds and dressed until the seeds showed a black color in a shade to avoid direct sun light so as to maintain the viability of the inoculums.

Flat sowing of two seeds per hill to a depth of 5 cm was done, and following establishment, the plots were thinned to one plant per hill. At the time of sowing, a blended NPSB fertilizer was applied in a band. Weeding was done by hand following the fourth and fifth weeks of sowing. To prevent Rhizobia cell death due to desiccation, the seeds were promptly covered with moist soil after sowing. From field preparation to harvesting, all relevant agronomic managements were carried out equally. On August 20, 2019, harvesting was completed when the pods turned a brownish red color.

### 2.5 Data collected

Data on total number of nodules and effective nodules were collected from five randomly selected plants grown in rows left for destructive sampling during flowering stage. The total and effective nodules were studied after washing the root samples carefully in plastic containers to remove the soils attached to the roots. The separated root nodules were counted, the total was recorded, and then the nodules were dissected using forceps and knives to determine the nodule effectiveness based on the nodules internal color. Hence, the nodules with pink or dark red internal color were recorded as effective and those nodules with white color were recorded as non-effective. Data on yield and yield component parameters were taken in each plot from five randomly selected plants at physiological maturity and at harvest time, respectively. Hundred seeds weight was determined from 100 randomly taken seeds from plants grown in the net plot area at harvest and the seeds weight was determined using a sensitive balance. Number of pods per plant, and number of seeds per pod were collected from five randomly selected plants in the net plot area at harvest. Aboveground dry biomass yield was measured from five plants left for destructive sampling at physiological maturity and converted into hectare basis. Grain yield was measured from the net plot area at harvest and adjusted to 10% moisture content. Harvest index (HI) was calculated as the ratio of grain yield to total aboveground biomass multiplied by hundred.

### 2.6 Statistical data analysis

The data were subjected to analysis of variance (ANOVA) using SAS version 9.1’s Generalized Linear Model, and interpretations were derived using procedures described on Gomez and Gomez [22]. The least significant difference (LSD) test was used to separate the means at a 5% level of significance.

### 2.7 Economic analysis

CIMMYT’s (1988) partial budget procedure was used to conduct an economic study. The variable costs included the blended NPSB fertilizer cost (14 ETB kg^−1^) and the cost of each Rhizobia strain type HB-A15 and *Rhizobia etli* (200 and 240 ETB ha^−1^, respectively), at the time of planting and their application, while the price of the current common bean was considered as gross benefit, according to the partial budget procedure described by CIMMYT [23]. The labor costs associated with inoculating common bean seeds with Rhizobia strains and applying NPSB fertilizer were documented and employed in this research as well. The average open market price (ETB kg^−1^) was used to appraise the common bean grain price.

## 3 Results and discussion

### 3.1 Physico-chemical properties of soil of experimental site

The soil physico-chemical parameters of the study site are indicated in Table 1. According to the soil analysis data, the soil texture class of the experimental location is clay loam (Table 1). The pH of the experimental site’s soil is in the moderately acidic range [24]. Similarly, according to Landon [24], the experimental site’s CEC is medium.

**Table 1:** Physico-chemical properties of soil

Soil physico-chemical property	Values
Texture	Clay loam
Sand (%)	59
Silt (%)	19
Clay (%)	22
Textural class	Clay loam
pH	5.8
Total N (%)	0.172
Available P (ppm)	1.54
CEC (cmol(+)/kg soil)	22.4
O.C (%)	2
Available S (ppm)	18.2
Available B (ppm)	0.42

### 3.2 Total and effective number of nodules

#### 3.2.1 Total number of nodules

Application of NPSB fertilizer and inoculation of Rhizobia strain had significant (*P* > 0.05) effect on the total number of nodules (Table 2). The Rhizobia strain type *Rhizobium etli* produced the highest number of total nodules (86.6), whereas the treatment without Rhizobia inoculation produced the lowest number of total nodules (44) (Table 3). When compared to HB-A15 Rhizobia strain type and control treatment, inoculation with *Rhizobium etli* resulted in a higher number of nodules per plant, which could be due to the fact that *Rhizobium etli* had better nodulation inducing capacity than HB-A15 and naturally pre-existing soil *Rhizobium* species. The presence of Rhizobia strain type specificity could potentially explain the result. Many researchers agreed that inoculating common beans with the right *Rhizobium* strain increases the number of nodules per plant compared to uninoculated beans [25,26,27,28]. Recent finding by Genetu et al. [11] and Ketema and Tefera [28] indicated significant increase in the number of nodule per plant for faba bean in different parts of Ethiopia due to Rhizobia inoculation. In addition, several researchers showed that inoculating common bean with efficient Rhizobia strains resulted in much higher nodulation and N_2_ fixation rates than the native Rhizobia population [29].

**Table 2:** Mean squares of ANOVA for yield and yield components of common bean as affected by Rhizobium inoculation and NPSB rate

Source of variation		Mean squares							
	Df	BM	GY	HI	NP	NS	HW	NTN	NEN
Block	2	38,260	130,853	319.26	3.096	0.17422	116.8	37.94	44.5
Bio-fertilizer	2	23,297^ns^	285,168**	1.99	157.818**	2.0009**	83.80**	722.04**	306.59*
NPSB rate	4	5,541,910**	2,647,396**	104.79**	27.879*	0.293^ns^	78.52**	7077.77**	3243.54**
BF xFR	8	314,495**	47,564*	1.812^ns^	3.48^ns^	0.1153^ns^	9.05^ns^	39.5^ns^	301.68*
Error	28	47,417	19,641	14.208	7.781	0.139	7.31	82.3	117.99
CV		4.01	5.67	8.68	12.14	8.83	9.43	13.41	20.49

Where, Df = Degree of freedom; ** = highly significant (*P* = 0.01); * = significant (*P* = 0.05); ns = non-significant; CV (%) = coefficient of variation; BM = above ground biomass; GY = grain yield; HI = harvest index; NP = number of pods; NS = number of seeds and HW = hundred seeds weight; NTN = number of total nodules; NEN = number of effective nodules.

**Table 3:** Mean number of total nodules, Number of total nodules per plant, Number of pods per plant, Number of seeds per plant, and Hundred seeds weight (g), HI of common bean as affected by main effect of blended NPSB fertilizer and Rhizobia strain type at Offa-sere kebele during 2019 main crop season

Rhizobia strain	Number of total nodules per plant	Number of pods per plant	Number of seeds per pod	HW (g)	HI
HB-A15	72.40^b^	24.57^a^	4.29^b^	28.24^b^	46.97^a^
*Rhizobium etli*	86.61^a^	25.13^a^	4.55^a^	31.23^a^	46.22^a^
Withoutinoculation	43.95^c^	19.25^b^	3.83^b^	26.56^c^	42.36^b^
LSD (0.05)	6.7856	2.10	0.28	2.02	2.25
CV (%)	13.41	12.14	8.83	9.43	6.65
Blended NPSB rate (kg ha^−1^)					
0	56.24^d^	20.38^b^	—	24.22^d^	38.31^c^
50	62.01^cd^	22.11^ab^	—	27.62^c^	41.71^c^
100	68.8^bc^	23.42^a^	—	28.98^bc^	52.36^a^
150	79.54^a^	24.42^a^	—	30.88^ab^	46.73^b^
200	71.69^ab^	24.59^a^	—	31.68^a^	46.82^b^
LSD (0.05)	8.76	2.69	—	2.61	2.90
CV (%)	13.41	12.14	—	9.43	6.65

Mean values in the table followed by the same letter(s) are not significantly different from each other at 5% level of significance, LSD (0.05) = Least significant difference at 5% level of significance; and CV (%) = coefficient of variation.

In the case of blended NPSB fertilizer, the number of total nodules increased as the blended NPSB fertilizer was increased only up to 150 kg NPSB ha^−1^. This probably indicates that applying NPSB fertilizer beyond 150 kg ha^−1^ may cause elemental N from blended NPS fertilizer to have an antagonistic effect on the crop root zone. Further, the increase in total nodule number with the increase in NPSB level even up to the indicated level might be attributed to better supply of P and S nutrients, which are important for nodulation. This finding is consistent with Chala et al. [30], who reported significant effect of NPS fertilizer application on total number of nodule per plant. Dereje et al. [31] also reported the significant effect of P application on total nodule number of common bean at Areka. On the contrary, Chen et al. [32] and Starling et al. [33] reported that a high nitrogen rate (56.58 kg N ha^−1^) reduced nodule quantity and weight in soya bean.

#### 3.2.2 Number of effective nodules

The number of effective nodules was significantly affected (*P* < 0.05) by the interaction of blended NPSB fertilizer and Rhizobia strain types (Table 2). The plot that received a combined application of 150 kg ha^−1^ of NPSB fertilizer with inoculation of *Rhizobium etli* had the largest number of effective nodules (75.13), whereas the control plot had the lowest (30) (Table 4). The probable reason for increase in the number of effective nodules could be due to the improved availability of nutrients such as P and S following application of NPSB, which are crucial for effective nodulation and the suitability of the *Rhizobium* strain for effective nodulation.

**Table 4:** Mean number of effective nodules of common bean as affected by interaction effect of blended NPSB fertilizer and *Rhizobia* strain at Offa-Sere kebele during 2019 main cropping season

	Blended NPSB rate (kg ha^−1^)				
Rhizobia strain	0	50	100	150	200
**Effective nodules**					
HB-A15	48.07^cde^	50.60^cd^	53.07^bcd^	64.83^abc^	57.07^abc^
*Rhizobium etli*	59.33^abc^	62.67^abc^	70.73^ab^	75.13^a^	70.70^ab^
Wi	29.93^ef^	31.47^ef^	27.93^f^	57.73^abc^	35.93^def^
LSD (0.05)			18.17		
CV (%)			20.49		
**Grain yield (kg ha** ^**−1**^)					
HB-A15	1,879^e^	2241.7^d^	2748.7^bc^	2923.7^ab^	2,922^ab^
*Rhizobium etli*	1747.7^e^	2291.7^d^	2887.3^ab^	3017.7 ^a^	2,867^ab^
Wi	1496.3^f^	1759.3^e^	2,622^c^	2770.3^bc^	2,924^ab^
LSD (0.05)			245.3		
CV (%)			5.67		
**Aboveground dry biomass yield (kg ha** ^**−1**^)					
HB-A15	4,607^h^	5,194^ef^	5183.7^ef^	6,326^b^	6096.7^bc^
*Rhizobium etli*	4566.7^h^	5060.3^fg^	5545.3^de^	6014.7^bc^	5,847^cd^
Wi	4,171^i^	4,776^gh^	5043.7^fg^	6,367^b^	6383.7^b^
LSD (0.05)			364.2		
CV (%)			4.01		

Mean values in the table followed by the same letter(s) are not significantly different from each other at 5% level of significance, LSD (0.05) = Least significant difference at 5%; and CV (%) = coefficient of variation; Wi = without inoculation.

This study is consistent with Genetu et al. [11], who observed highly significant (*P* < 0.001) greater nodulation by the interaction effect of the inoculant and blended fertilizer. This may be attributed to the positive effect of sulfur in nodulation as it plays a vital role in biosynthesis of nitrogenase, which is employed in nitrogen fixation, in common beans Becana et al. [34]. Chala et al. [30] also reported significant (*P* < 0.01) effect of *Rhizobia inoculation* and NPS on the number of effective nodules per plant. Tsai et al. [35] found that applying N at a rate of 22–33 kg ha^−1^ increased both the nodulation and seed output in French bean (*P*. *vulgaris*).

### 3.3 Yield and yield components

#### 3.3.1 Number of pods per plant

The Rhizobia strain and the application of blended NPSB fertilizer had significant effect on the quantity of pods per plant (Table 2). The maximum number of pods per plant was observed after inoculation with *Rhizobium etli*, which is statistically equal to HB-A15, and the lowest number of pods per plant was observed after no inoculation (Table 3).

As a result, the increase in pods per plant could be attributed to improved vegetative growth [28] following effective nodulation with the new Rhizobia strains (*Rhizobium etli* and HB-A15). Inoculation with *Rhizobium etli* also increased the number of pods per plant, which could be attributable to the novel strain’s efficacy in nodulation and N-fixation over native Rhizobia in the experimental soils. This conclusion is supported by studies from Ethiopian and international scholars [36,37,38]. According to Yoseph and Shanko [36], inoculation with Rhizobium strain HB-429, for example, enhanced the number of pods per plant of common bean substantially over the control.

Improved number of pods per plant in legumes was also ascribed to appropriate supply of important plant nutrients such as N [36], P [39], and/or NPS [36,40]. For example, Deresa et al. [40] suggested that the increased number of pods per plant for common bean with increased NPS rates could be due to adequate N, P, and S availability, which could have facilitated the production of primary branches and plant height, resulting in the production of a higher number of total pods.

Confirming these finding different researchers found a considerable increase in the number of pods per plant of common bean in the study area or elsewhere in the country following application of NPS/NPSB blended fertilizer [41, 42 43]. The increase in number of pods per plant due to NPSB fertilizer application might be attributed to better supply of other nutrients such as S, P, and B from blended NPSB fertilizer, which leads to improved dry matter accumulation, growth, and yield component formation [36]. For example, Shumi [42] found that at a rate of 250 kg NPS ha^−1^, the largest number of pods per plant was recorded, whereas the unfertilized plot had the lowest number of total pods.

#### 3.3.2 Hundred seed weight

The results also demonstrated that the Rhizobia strain and blended NPSB fertilizer application had a substantial impact on HW (Table 2). The plot that was inoculated with the HB-429 type of Rhizobia strain had the highest HW, while the plot without inoculation had the lowest HW (Table 3). Increased nodulation and N bio-availability for absorption and assimilation, resulting in improved vegetative growth and re-translocation of photo-assimilates for grain filling, could explain the increase in HW. This also suggests that the Rhizobia strain HB-429 is more efficient than the native Rhizobia strain, which has a paramount importance improving nodulation and growth. Improved growth and N-fixation can lead to higher grain filling by allowing assimilates to be translocated later in the growth cycle, resulting in a positive contribution to seed weight. Corroborating the current finding, different authors have found considerable increases in HW of common bean [44], chickpea [37], and faba bean [28] after inoculation with Rhizobium bacteria. Girma et al. [44] also found that Rhizobium inoculation of haricot bean influenced thousand seed weight strongly and significantly (*P* < 0.001) with the highest thousand seed weight (162.95 g) obtained at Rhizobium inoculation treatment.

Furthermore, due to the use of blended NPS fertilizer, the HSW varied greatly. As a result, the greatest NPS fertilizer rate yielded the highest HW, while the lowest yielded the smallest (Table 3). The increase in HWs might be due to the direct supply of N, P, S, and B from inorganic mixed NPSB, which are critical for plant growth and development, boosting photosynthetic efficiency and dry-matter accumulation, and resulting in greater grain filling. Different researchers observed a substantial influence of blended NPS [40] or NPKSB fertilizer [43] on HW of common bean, which is consistent with the current findings. For example, Abebe and Mekonnen [43] found that increasing the rate of blended NPKSB fertilizer application had a substantial impact on common bean HW.

#### 3.3.3 Number of seeds per pod

The number of seeds per pod was considerably affected by Rhizobia inoculation (Table 2). The plot with *Rhizobia etli* produced the maximum number of seeds per pod, while the plot with no inoculation produced the lowest number of seeds per pod (Table 3). This can be explained by the fact that *Rhizobia* strain inoculation can improve symbiotic N fixation and N nutrition, which in turn enhance assimilate production, thereby promoting growth, which can be re-translocated for the formation of yield containers such as seeds, thus increasing the number of seeds per pod. Corroborating the results, different authors reported significant effect of *Rhizobium* inoculation on number of seeds per pod for different legumes common bean [36] and faba bean [28,45,46]. Yoseph and Shanko [36] showed a remarkable 115% increase in the number of seeds per pod of common bean inoculated with *Rhizobium* bacteria as compared to the control. Recent study by Ketema and Tefera [28] also reported highly significant (*P* < 0.001) effect of *Rhizobium* rate on the number of seeds per pod of faba bean in Southern Ethiopia. Furthermore, Samuel et al. [45] reported that *Rhizobia* inoculation resulted in considerable increases in the number of seeds per plant for faba bean.

#### 3.3.4 Grain yield

The interaction effect of Rhizobia strain and blended NPSB fertilizer on grain yield was significant (*P* < 0.05) (Table 2). Inoculation of *Rhizobium etli* with application of 150 kg ha^−1^ blended NPSB produced the highest grain yield, whereas the control produced the lowest grain yield (Table 4). This means that both new *Rhizobia* strain inoculation and inorganic NPSB application can alleviate the soil fertility problem (Table 1), which is a direct cause of poor production in the study location. In other words, enhanced grain production with increased blended NPSB fertilizer rate and *Rhizobia* inoculation could be attributed to increased availability of nutrients like P, S, and B because of either blended NPSB fertilizer application or improved N-fixation by *Rhizobia* strain. Regardless of this, increasing the level of N along with the rate of NPSB might interfere with the atmospheric N-fixation. Higher availability of these nutrients is important for growth, development, and assimilates production [47], which leads to increased grain production via photo-assimilates being re-translocated from the vegetative to the yield components. The results of this study are supported by the findings of different authors [27,41,48].

For instance, Endrias [41] found the maximum grain yield (2,257 kg ha^−1^) from the interaction of *Rhizobium* inoculation with 100 kg ha^−1^ NPS fertilizer treatment, and the lowest grain yield (665 kg ha^−1^) from the plot that got no blended NPS fertilizer application. Abera and Abebe, [27] reported significantly higher mean seed yield for field as a result of interaction of 125 kg NP ha^−1^ and *Rhizobium* inoculation at Gedo, Oromia region, Ethiopia. Abera and Tadele [48] also found that combining *Rhizobia* strains with nitrogen had a substantial impact on grain yield of common bean in the Areka district, which is close to the study location.

#### 3.3.5 Aboveground dry biomass

The aboveground dry biomass yield of common bean was significantly affected (*P* < 0.05) by the interaction effect of blended NPSB fertilizer and *Rhizobia* strain types (Table 2). The maximum aboveground dry biomass (6383.7 kg ha^−1^) was found in plots that received a blended NPSB rate of 200 kg ha^−1^ without inoculation, which is also statistically at par with plots that receive 150 kg NPSB ha^−1^ along with inoculation of HB-A15 *Rhizobia* strain, while the lowest (4,171 kg ha^−1^) was found in plots applied with nil rate of NPSB along with no inoculation (Table 4). The improved availability of N and other nutrients P, S, and B directly from the applied inorganic fertilizer might have significantly increased the vegetative growth of the crop, which in turn could be attributed to the increase in biomass yield of common bean. This also indicates that the native *Rhizobia* strain is more effective than the new *Rhizobia* strain in enhancing vegetative growth. Endrias [41], also reported highest (5,006 kg ha^−1^) aboveground dry biomass from *Rhizobium* inoculation along with NPS rate at 100 kg ha^−1^. Veeresh [49] also found total dry matter production per plant increased significantly from 12.0 to 16.03 g as a result of increasing the rate of N from 40 to 120 kg ha^−1^ on French bean (*Phaseolus vulgaris*). The findings of this study are also backed up by Yoseph and Shanko [36], who found a considerable favorable effect of N fertilizer and *Rhizobium* application on shoot dry weight.

#### 3.3.6 HI

The results showed that the rate of blended NPSB fertilizer and the *Rhizobia* strains had a significant (*P* < 0.05) impact on the HI (Table 2). The plot with the highest fertilizer rate of 100 kg ha^−1^ had the highest HI, whereas the plot with the lowest fertilizer rate had the lowest HI (Table 3). According to this research, the favorable effect of NPSB fertilizer on nutrient and photo-assimilate re-translocation from the vegetative to grain parts of the crop may explain the improvement in HI with increase in the blended NPSB fertilizer rate. Birhan [50] also recorded a considerable rise in HI with the application of P on common bean, which is consistent with this finding. Further, Chala et al. [30] reported significant effects of NPS on HI of soybean. The increase in HI with fertilizer rates is also consistent with Dhanjal et al. [51], who found that raising N levels from zero to 60 and 120 kg N ha^−1^ improved HI values by 31.60, 31.99, and 33.86%, respectively. In contrast, Chala et al. [30] reported non-significant effect of blended fertilizer application on HI of faba bean.

Furthermore, when a crop was inoculated with a new *Rhizobia* strain, the HI increased. The reason for this could be because inoculation increased N bioavailability, absorption, and assimilation through biological N-fixation, which boosted crop growth and partitioning of dry matter to grain. Inoculation of *Rhizobium etli* and without inoculation had the highest (46.97%) and lowest (42.36%) percent of HI, respectively. Corroborating the findings, both Anteneh and Bulti [52] on common bean and Chala et al. [30] on Soybean observed significant effect of seed inoculation on HI of both crops.

### 3.4 Economic analysis

According to the economic analysis, the NPSB fertilizer rate of 100 kg ha^−1^ combined with inoculation of *Rhizobia* strain, *Rhizobium etli* gave economically optimum net return (37238.955 ETB ha^−1^) with the maximum marginal rate of return of 4579.77% followed by 38298.5 ETB with 3,702% marginal rate of return attained at 100 kg NPSB ha^−1^ along with inoculation of the same Rhizobium strain (Table 5). This finding suggests that applying 150 kg ha^−1^ of NPSB with the *Rhizobium etli* yields a good economic return in the research area and other places with similar agro-ecological parameters (Table 5). In agreement, Kiros and Atsede [53] reported that *Rhizobium* inoculation along with 125 kg NPSB ha^−1^ gave the highest net benefit with acceptable marginal rate of return.

**Table 5:** Partial budget analysis of the responses of common bean to Rhizobia inoculation and NPSB fertilizer application rates

Rhizobium inoculation	NPSB kg ha^−1^	UGY kg ha^−1^	AGY (kg ha^−1^)	GFB (ETB ha^−1^)	TVC (ETB ha^−1^)	NB (ETB ha^−1^)	MRR (%)
HB-A15	0	1,879	1691.1	25366.5	200	25166.5	
*Rhizobium etli*	0	1747.67	1572.903	23593.55	240	23353.545	D
Wi	0	14946.33	13451.697	201775.45	0	201775.455	D
HB-A15	50	2241.67	2017.503	30262.55	1,000	29262.545	D
*Rhizobium etli*	50	2291.67	2062.503	30937.55	1,040	29897.545	1587.5
Wi	50	1759.33	1583.397	23750.95	800	22950.955	2894.41
HB-A15	100	2748.67	2473.803	37107.05	1,700	35407.045	1384.01
*Rhizobium etli*	100	2887.33	2598.597	38978.95	1,740	37238.955	4579.77
Wi	100	2,622	2359.8	35,397	1,500	33,897	1392.46
HB-A15	150	2923.67	2631.303	39469.55	2,400	37069.545	352.51
*Rhizobium etli*	150	3017.67	2715.903	40738.55	2,440	38298.545	3072.5
Wi	150	2770.33	2493.297	37399.455	2,200	35199.455	1291.29
HB-A15	200	2,922	2629.8	39,447	3,100	36347	127.51
*Rhizobium etli*	200	2,867	2580.3	38704.5	3,140	35564.5	D
Wi	200	2,924	2631.6	39,474	2,900	36,574	420.63

Where, UGY = unadjusted grain yield; AGY = adjusted grain yield; GFB = gross field benefit; TVC = total variable costs; NB = net benefit; MRR = marginal rate of return; ETB/ha = Ethiopian Birr per hectare; D = dominated treatments.

## 4 Conclusion

The results of the present study showed that common bean production in the study site is constrained by nutrient deficiency. Hence, single application of either blended NPSB fertilizer or inoculation of Rhizobia strain or application of both together can correct soil fertility problems and increase yield and yield attributing traits of common bean. Accordingly, common bean grain yield was increased with increased rate of blended NPSB fertilizer and Rhizobia strain inoculation. Based on the results of this study, it could be tentatively concluded that inoculation of *Rhizobium etli* strain along with application of NPSB at the rate of 100 kg ha^−1^ was found to be the appropriate treatment combination from agronomic and economic point of view for common bean production in the area and other areas with similar agro-ecologies.
